# Supplementary material for: Seismic evidence for a mantle suture and implications for the origin of the Canadian Cordillera
Source: Nat Commun. 2019 May 21;10:2249. doi: 10.1038/s41467-019-09804-8 (PMC6529410; doi:10.1038/s41467-019-09804-8)
Supplement: Supplementary file 1 — Supplementary Information [file 41467_2019_9804_MOESM1_ESM.pdf]

Seismic evidence for a mantle suture and implications for the origin of the Canadian Cordillera

Chen et al.

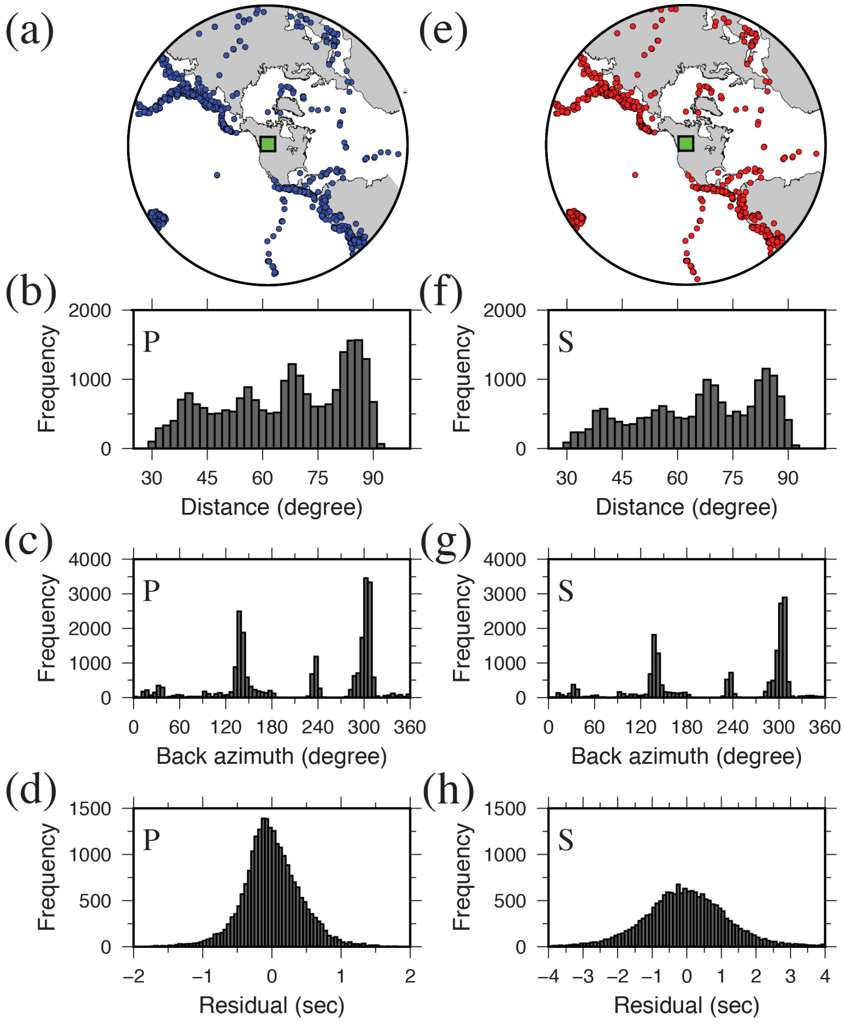

Supplementary Figure 1 Earthquake and travel-time data distributions. **(a)** Earthquake locations of P-wave data. Epicenters are clustered in the northwestern pacific and western coast of South America. The green square marks the region of study. **(b)** The distributions of epicentral distances. **(c)** The distributions of back azimuth. It shows a multimodal pattern concentrated between 120-180, 230-250 and 260-320 degrees. **(d)** The distributions of relative travel-time residuals. **(e)-(h)** The same as **(a)-(d)** but for S-wave data.

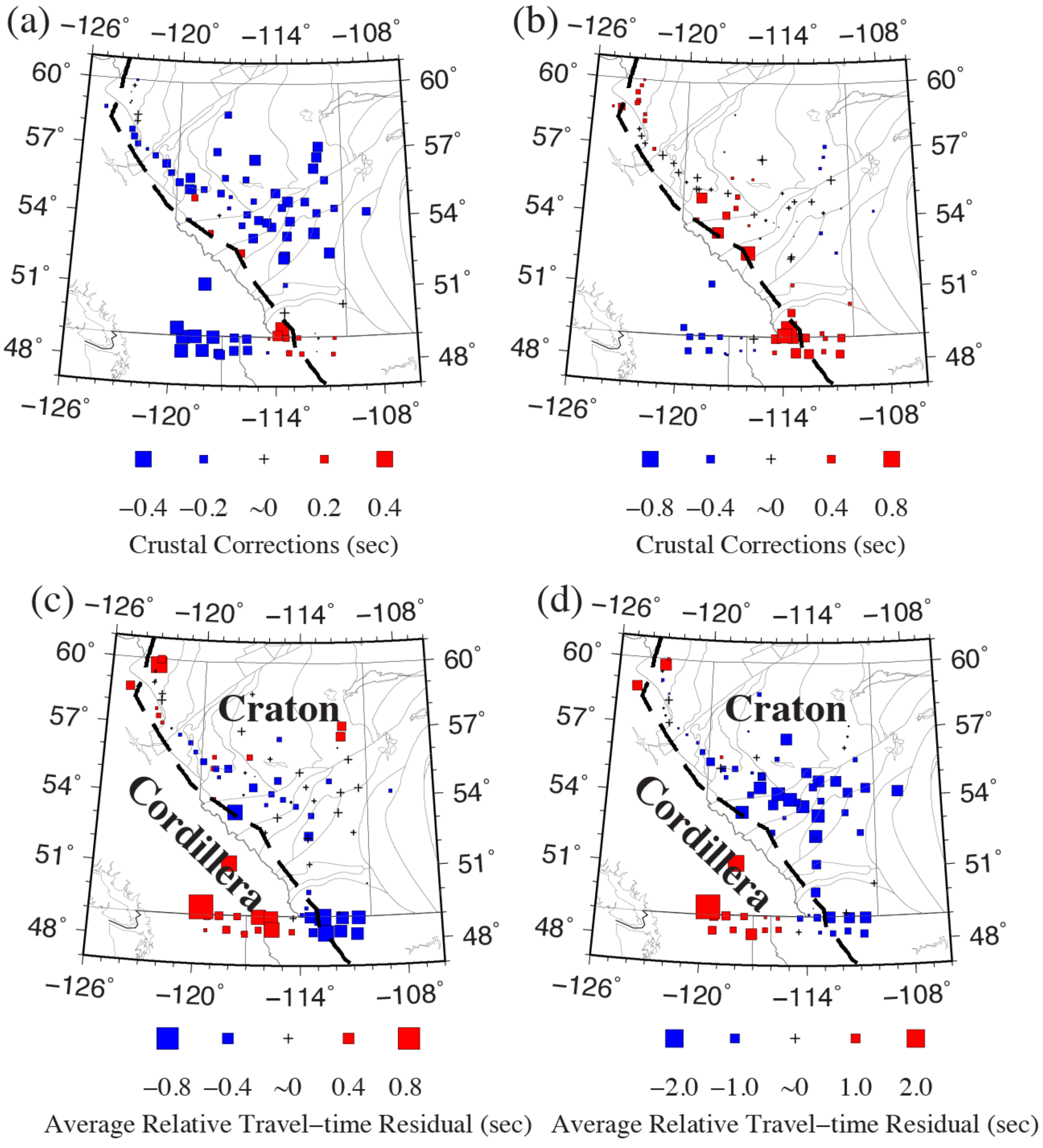

Supplementary Figure 2 P- and S-wave travel time measurements. **(a)** Average crustal correction term at each station. Blue and red colors represent negative and positive time corrections, respectively. The dashed line marks the Cordilleran Deformation Front. **(c)** Average relative travel-time residual after topographic and crustal corrections at each station. **(b)** and **(d)** are the same as **(a)** and **(c)** but for S-wave data.

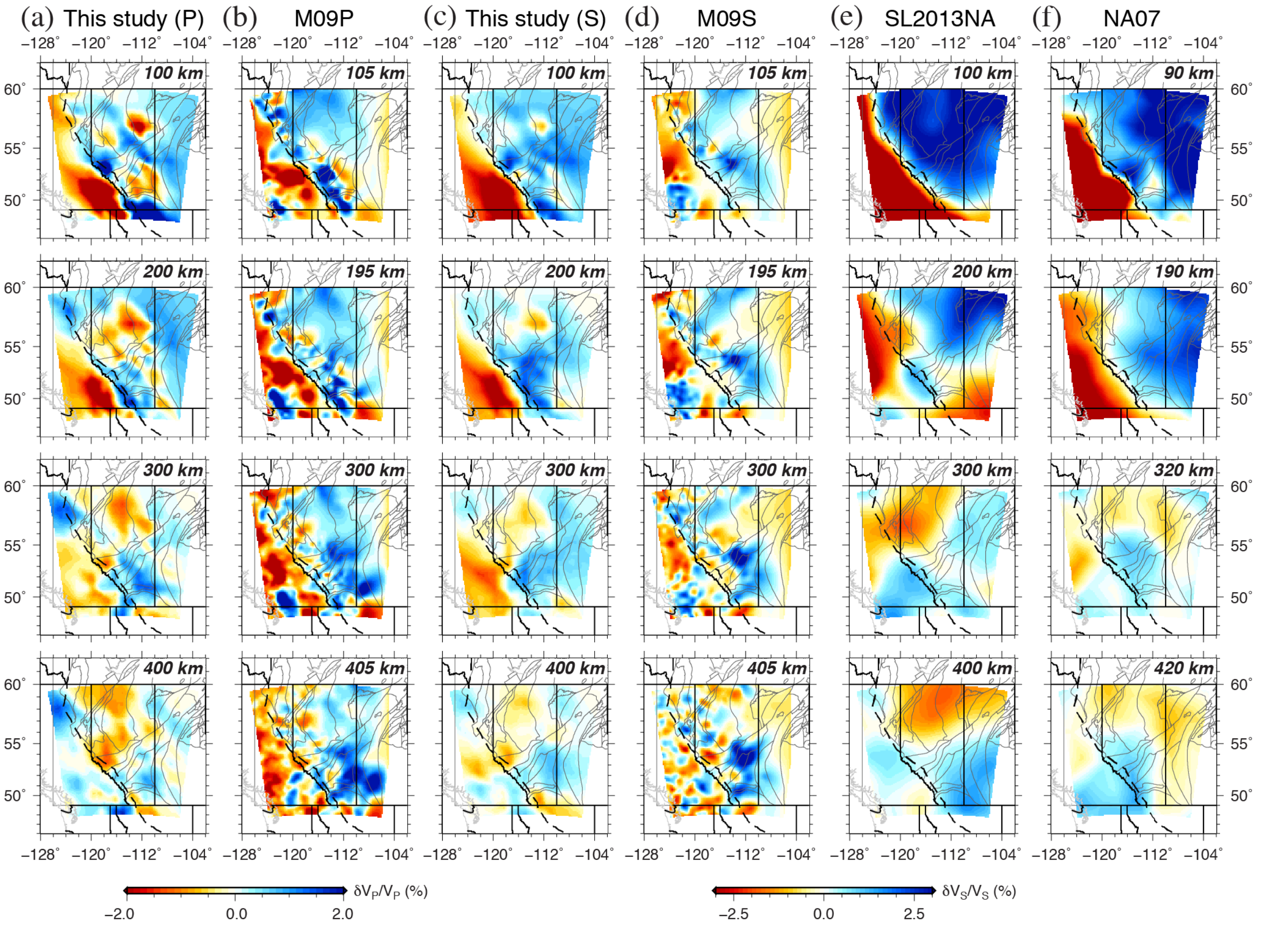

Supplementary Figure 3 Model comparison of six tomographic models at depths of 100-400 km. **(a)** P-velocity model from this study. **(b)** P-velocity model of western Canada from Mercier et al. (2009) (hereafter M09P)[1]. **(c)** S-velocity model from this study. **(d)** S-velocity model of western Canada from Mercier et al. (2009) (hereafter M09S)[1]. **(e)** S-velocity model of North America from Schaeffer and Lebedev (2014) (hereafter SL2013NA)[2]. **(f)** S-velocity model of North America from Bedle and van der Lee (2009) (hereafter NA07)[3].

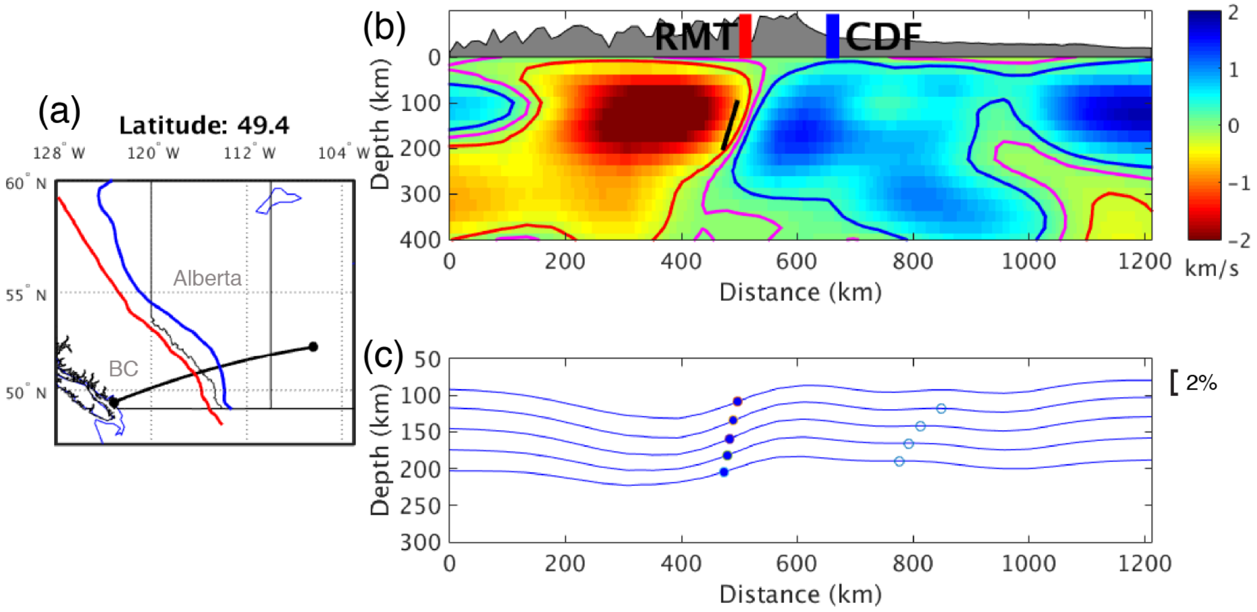

Supplementary Figure 4 The determination of Cordillera-Craton boundary location from velocity gradient measurement. **(a)** The location of a cross-section used for the measurement. The blue and red lines indicate the location of the Cordilleran Deformation Front (CDF) and Rocky Mountain Trench (RMT), respectively. **(b)** P-wave velocity perturbations along the cross-section shown in **(a)**. The red, purple and blue lines show -0.5, 0 and 0.5% velocity contours, respectively. The thick black line indicates the determined boundary location. The locations of the Cordilleran Deformation Front and Rocky Mountain Trench are marked at the surface. **(c)** The 1D horizontal velocities computed in depth range of 100-200 km using a 50 km depth running average. Circles on polynomials indicate the location of local maxima in velocity gradient. The solid circles indicate the selected boundary points. The scale length of 2% velocity perturbation is indicated. The y axis only shows approximate depths of these profiles.

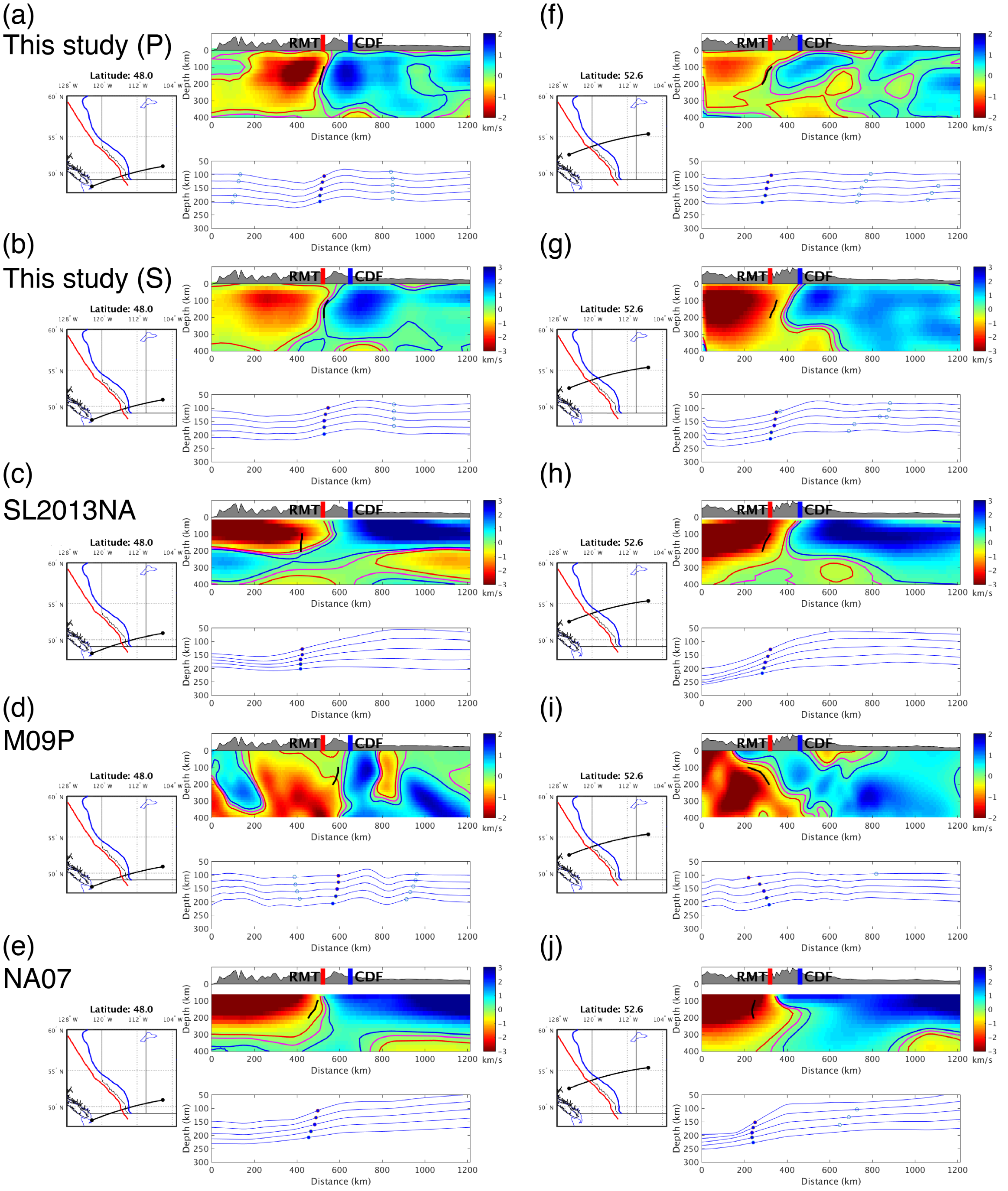

Supplementary Figure 5 Measurements of boundary locations from five different models along two profiles. **(a)** P velocity models from this study. **(b)** S velocity models from this study. **(c)** S-velocity model of North America from Schaeffer and Lebedev (2014) (SL2013NA)[2]. **(d)** P-velocity model of western Canada from Mercier et al. (2009) (M09P)[1]. **(e)** S-velocity model of North America from Bedle and van der Lee (2009) (NA07)[3]. **(f-j)** The same as **(a-e)** but for the northern profile.

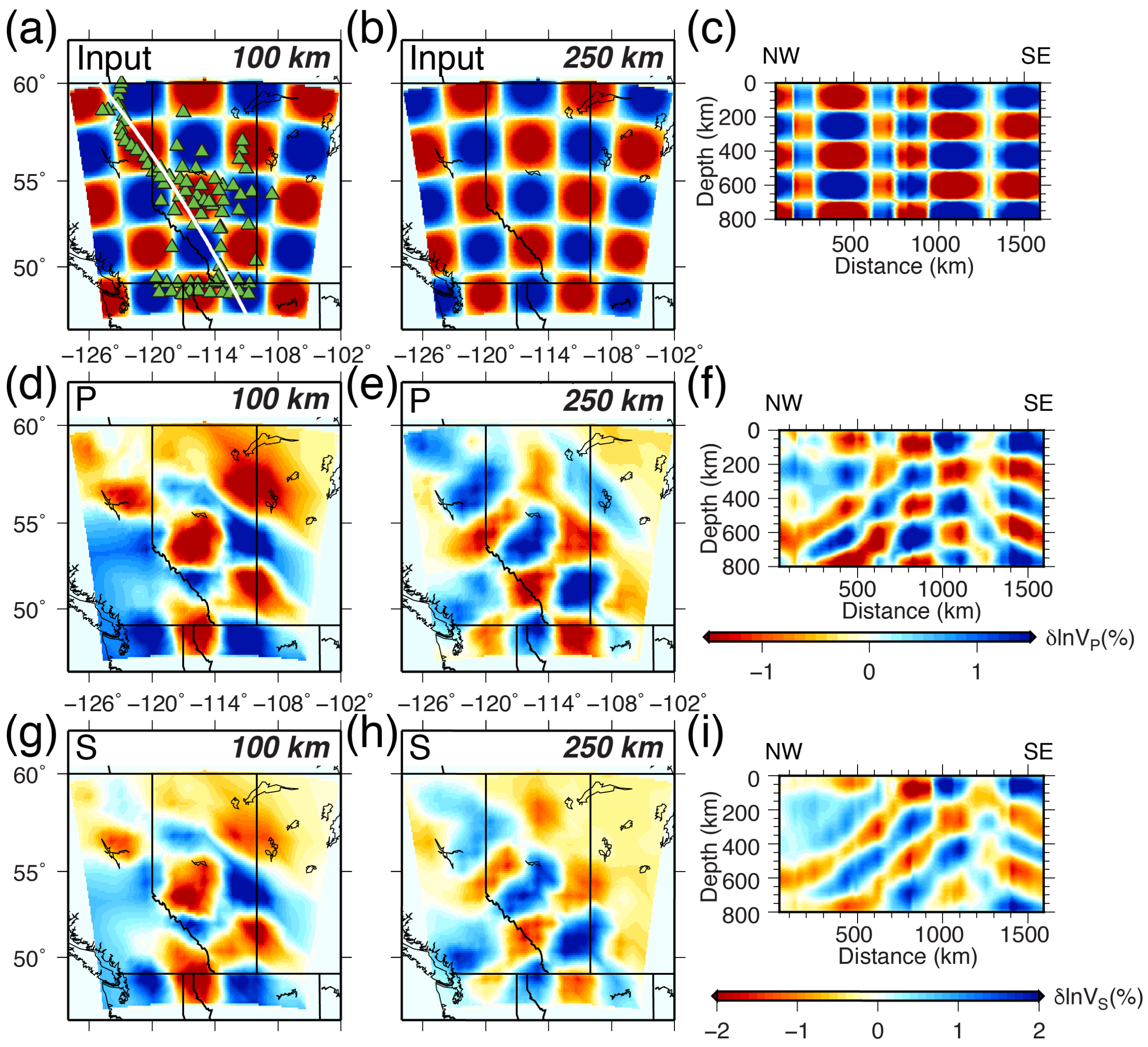

Supplementary Figure 6 Checkerboard resolution test of tomographic model. Input velocities at **(a)**100 km and **(b)** 250 km depths, and **(c)** along a cross-section. The profile location is marked by the white line at 100 km depth. The green triangles indicate the seismic stations. **(b-f)** Recovered P-wave velocities at the corresponding depths and along the same cross-section. **(g-i)** Recovered S-wave velocities at the corresponding depths and along the same cross-section.

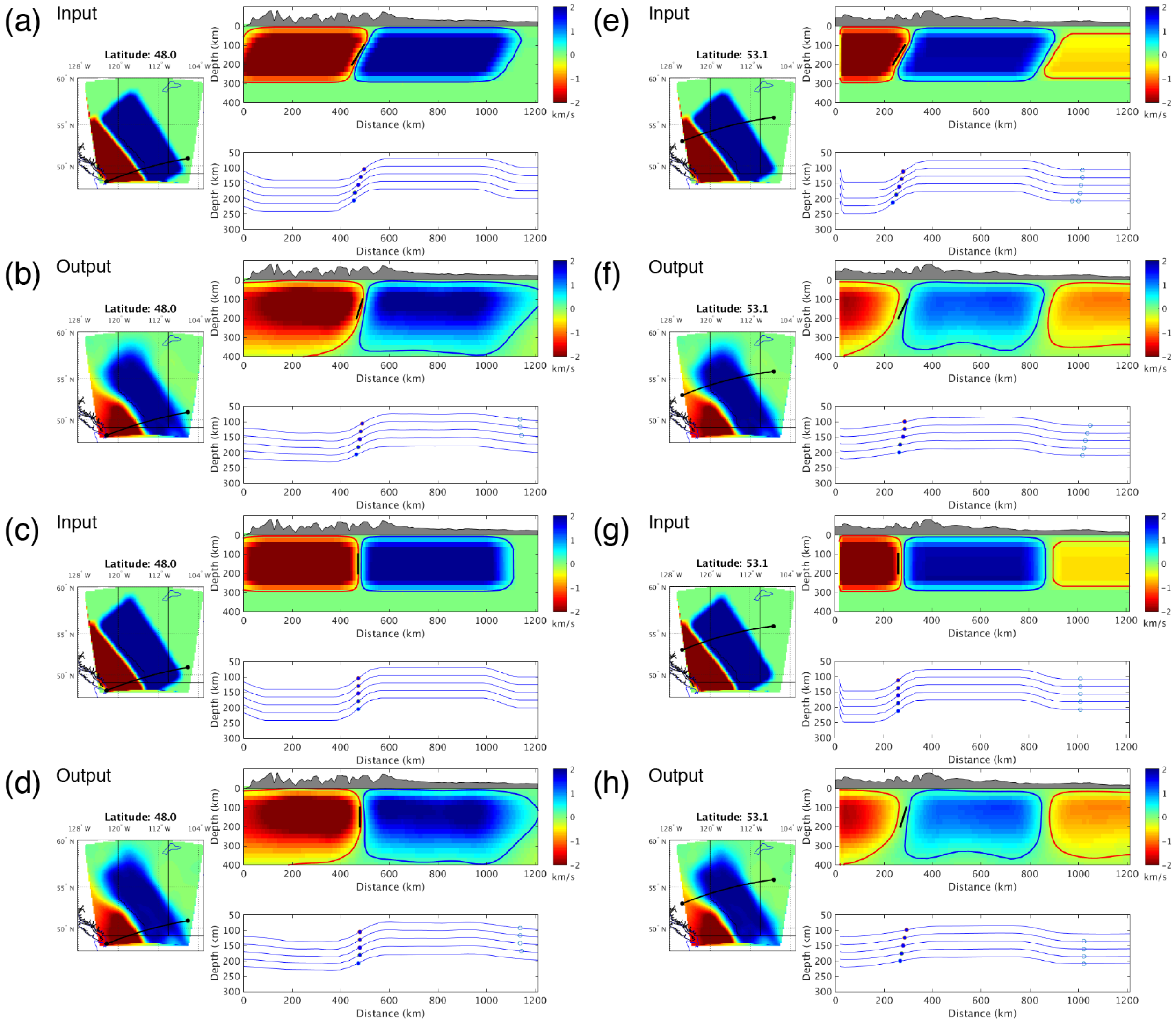

Supplementary Figure 7 Tests of the robustness of the boundary determination algorithm with a hypothetical input P-wave model that contains a dipping or vertical boundary. **(a)** Input and **(b)** output models with a dipping boundary. **(c)** Input and **(d)** output models with a vertical boundary. **(e)-(h)** The same as **(a)-(d)**, but for a cross-section in the north. In each plot, the location of profile is indicated by the black line on the map. The boundary location is determined from the maximum velocity gradient marked by the blue circles on the velocity profile.

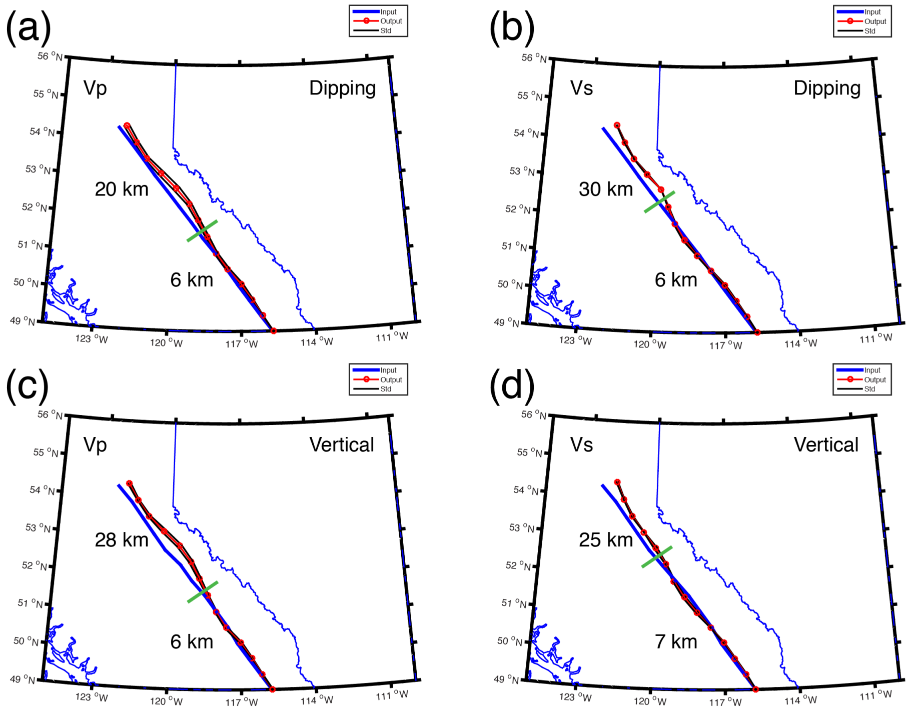

Supplementary Figure 8 The uncertainty in the boundary determination for P and S velocity models. Test results of P velocity model with a **(a)** dipping and **(c)** vertical boundary. The input and output boundary locations are shown by the blue and red lines, respectively. The uncertainty in the output boundary location is indicated by the black line defined as the standard deviation of the results from inversions with different damping parameters. The number labeled show the average discrepancy for northern and southern segments divided by the green bars. **(c)** and **(d)** The same as **(a)** and **(b)** but for the S velocity model.

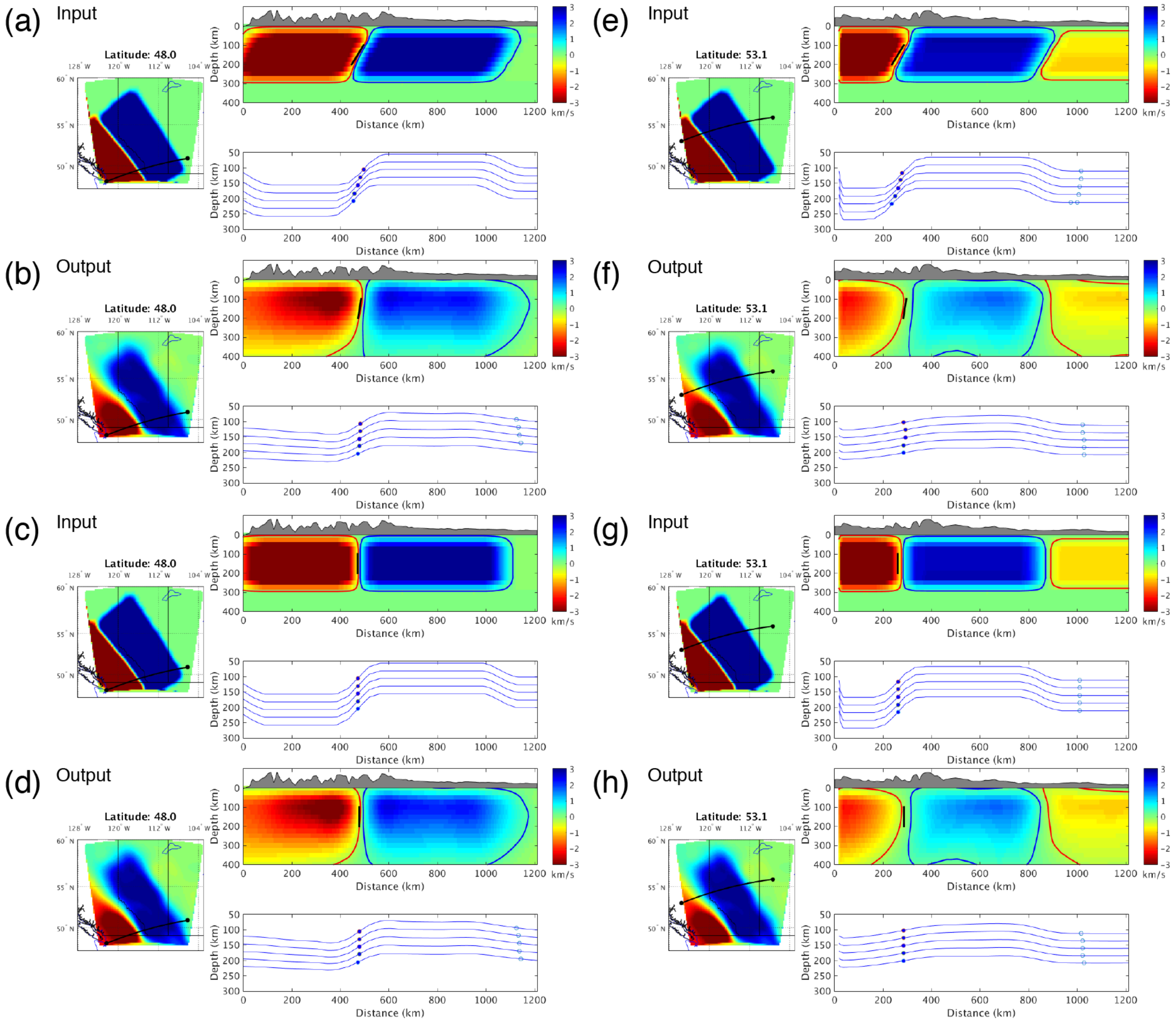

Supplementary Figure 9 Tests of the robustness of the boundary determination algorithm with a hypothetical input S-wave model that contains a dipping or vertical boundary. **(a)** Input and **(b)** output models with a dipping boundary. **(c)** Input and **(d)** output models with a vertical boundary. **(e)-(h)** The same as **(a)-(d)**, but for a cross-section in the north. In each plot, the location of profile is indicated by the black line on the map. The boundary location is determined from the maximum velocity gradient marked by the blue circles on the velocity profile.

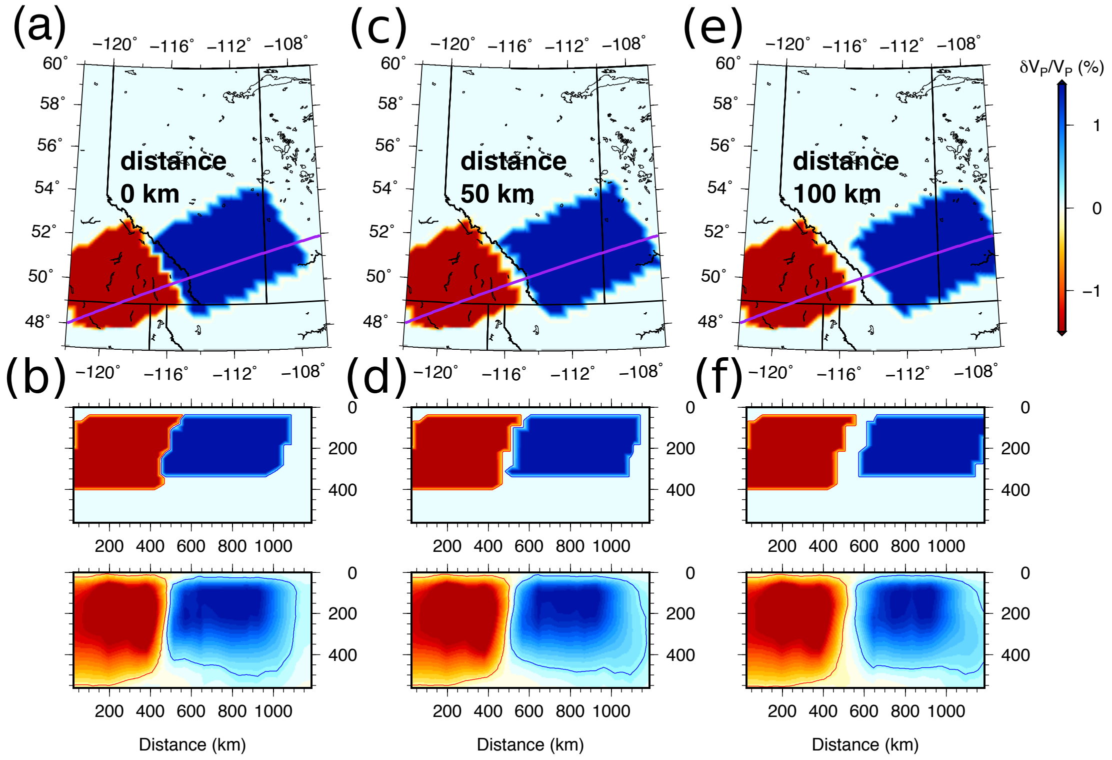

Supplementary Figure 10 Hypothesis tests with varying separation distances (labeled) between low and high velocity anomalies. **(a)** Input model at 100 km depth. **(b)** Input and output models along a cross-section shown in **(a)**. Tests for **(c)-(d)** 50 km and **(e)-(f)** 100 km separation distances.

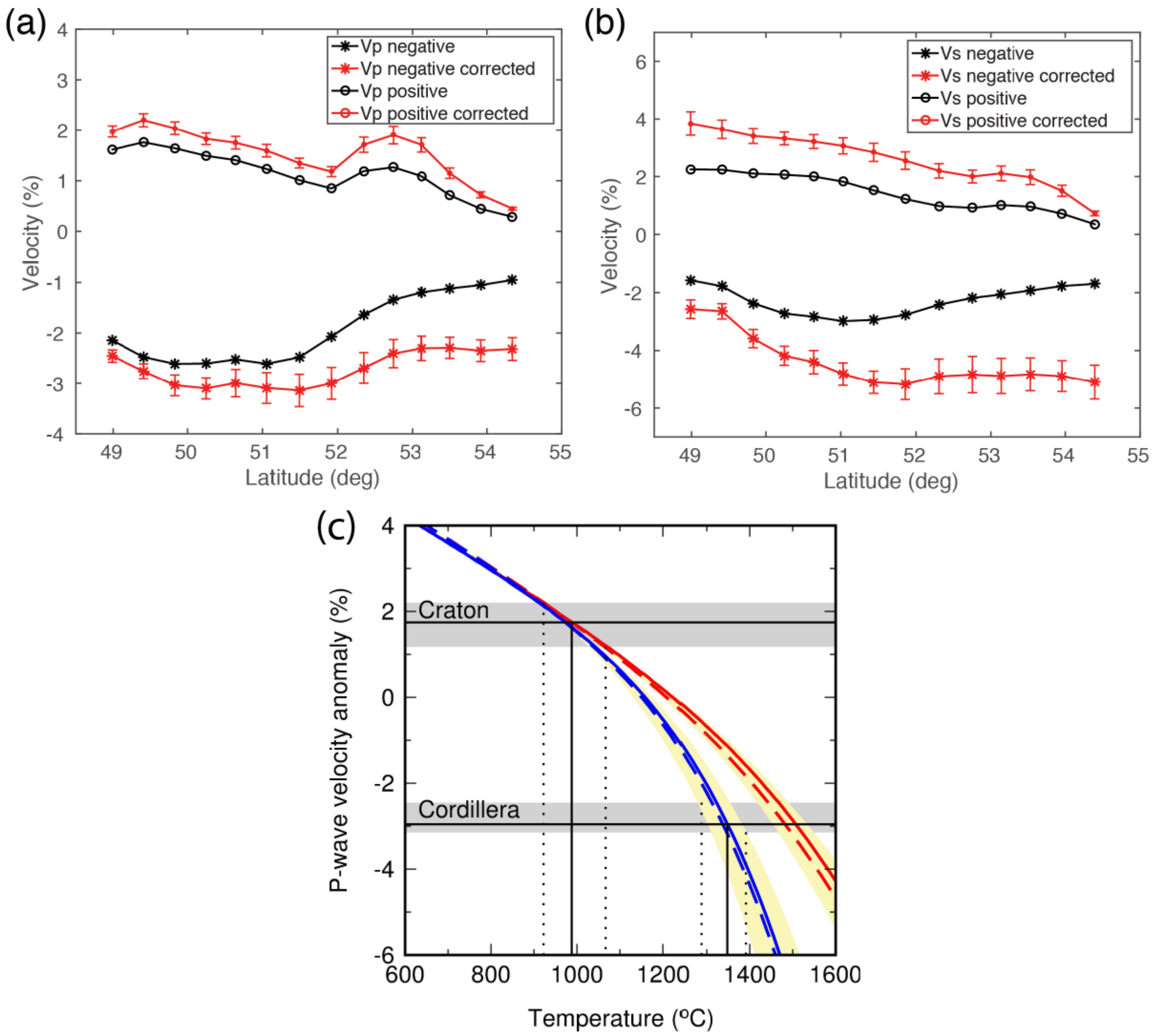

Supplementary Figure 11 Measurements of peak velocities on either side of the transition boundary for P and S velocity models. **(a)** Measurements for P velocity model. The black lines show the raw measurements from the model. The red lines show the corrected values after compensating for the amplitude underestimate based on the results of hypothesis tests. The error is defined as the standard deviation of the results from inversions with different damping parameters. **(b)** Measurements for S velocity model. **(c)** P-wave velocity-temperature (Vp-T) relationship for the Cordillera and craton regions. The horizontal black lines show the average P velocities with the corresponding uncertainties shaded in grey. The solid and dashed curves represent pyrolite (fertile) and dunite (depleted) compositions, respectively, in a dry (50 ppm H/Si;

red) and wet (5000 ppm H/Si; blue) mantle. The yellow shaded regions show the variations of Vp-T curves for various frequencies (0.03 to 2 Hz) and grain sizes (0.03 to 3 cm).

Supplementary References

1. Mercier, J. P. *et al.* Body-wave tomography of western Canada. *Tectonophysics* **475,** 480–492 (2009).
2. Schaeffer, A. J. & Lebedev, S. Imaging the North American continent using waveform inversion of global and USArray data. *Earth Planet. Sci. Lett.* **402,** 26–41 (2014).
3. Bedle, H. & van der Lee, S. S velocity variations beneath North America. *J. Geophys. Res. Solid Earth* **114,** (2009).
